# Genetic risk variants for childhood nephrotic syndrome and corticosteroid response

**DOI:** 10.3389/fped.2023.1248733

**Published:** 2023-10-06

**Authors:** Rachel K. Cason, Eileen Chambers, Tiffany Tu, Megan Chryst-Stangl, Kinsie Huggins, Brandon M. Lane, Alejandro Ochoa, Annette M. Jackson, Rasheed A. Gbadegesin

**Affiliations:** ^1^ Department of Pediatrics, Division of Nephrology, Duke University Medical Center, Durham, NC, United States; ^2^ Computational Biology and Bioinformatics Program, Duke Center for Statistical Genetics and Genomics, and Department of Biostatistics and Bioinformatics, Duke University, Durham, NC, United States; ^3^ Department of Surgery, Duke University Medical Center, Durham, NC, United States

**Keywords:** nephrotic syndrome, SRNS, SSNS, risk factors, genetics

## Abstract

**Introduction:**

The etiology of most cases of nephrotic syndrome (NS) remains unknown, therefore patients are phenotypically categorized based on response to corticosteroid therapy as steroid sensitive NS (SSNS), or steroid resistant NS (SRNS). Genetic risk factors have been identified for SSNS from unbiased genome-wide association studies (GWAS), however it is unclear if these loci are disease risk loci in other forms of NS such as SRNS. Additionally, it remains unknown if these risk loci are associated with response to therapy. Thus, we investigated the association between SSNS risk loci and therapy response in a large, multi-race cohort of children along the entire spectrum of childhood-onset NS.

**Methods:**

We enrolled 1,000 patients with childhood-onset NS comprised of SSNS and SRNS. Genotyping was done using TaqMan and Direct Sanger Sequencing for 9 previously reported childhood SSNS risk loci. We compared the allele frequencies (AF) and variant burden between NS vs. controls and SRNS vs. SSNS.

**Results:**

All 9 risk loci were associated with NS compared with healthy controls (*p* = 3.5 × 10^−3^–<2.2 × 10^−16^). Variant burden greater than 7 was associated with risk of SRNS (OR 7.4, 95% CI 4.6–12.0, *p* = 8.2 × 10^−16^).

**Conclusion:**

Our study showed that genetic risk loci for childhood SSNS are associated with pattern of therapy response, may help predict disease outcome, and set the stage for individualized treatment of NS.

## Introduction

Childhood nephrotic syndrome (NS) is a common condition seen in pediatric nephrology clinics and is a major cause of chronic kidney disease (CKD) in children. It is characterized by massive proteinuria, hypoalbuminemia, edema, and dyslipidemia. The prevalence of NS differs by phenotype, race, and geographic location (1). Corticosteroids are the mainstay of treatment for NS, and response to corticosteroid therapy is an important predictor of disease outcome (2). Patients with steroid sensitive NS (SSNS) often have excellent outcomes and rarely progress to kidney failure (1). Conversely, patients with steroid resistant NS (SRNS) frequently require intensified immunosuppression with agents other than corticosteroids in order to delay disease progression. Despite intensive immunosuppression, 50% of patients with SRNS will develop kidney failure within 5–10 years of diagnosis (3).

The etiology of childhood NS remains unclear; however, our knowledge of disease pathogenesis continues to expand. Pathogenic variants in >80 genes that are enriched in the podocyte have been associated with SRNS in 10%–30% of cases (4). A majority of SSNS are presumed to be due to immune dysregulation, possibly mediated by environmental factors and genetic variations in adaptive immunity genes (5). Several studies have identified genetic variants in adaptive and non-adaptive immunity genes, including *HLA-DQA1, BTNL2, HLA-DR/DQ, CALHM6, NPHS1/KIRREL, TNFSF15,* and *TNFRSF11A* (6–10). In these studies, only children with SSNS were included and did not include patients with SRNS. Thus, it is unclear if these loci are disease risk loci in SRNS. Additionally, it remains unknown if these risk loci are also associated with corticosteroid therapy response. The objective of this study is to determine the association between known SSNS genetic risk loci and pattern of corticosteroid therapy response in a large cohort of patients with both SSNS and SRNS. Defining this association is an important step in individualizing therapy for NS to minimize the significant side effects associated with exposure to multiple immunomodulatory agents.

## Methods and materials

### Patients

We enrolled 1,000 patients in a multi-race cohort with childhood-onset NS comprised of patients with SSNS (*n* = 639), and SRNS (*n* = 236), and unknown final therapy response (*n* = 125). Inclusion criteria were: (1) diagnosis of NS as defined by ≥2 + on urine dipstick or spot urine protein-to-creatinine ratio of ≥2 mg/mg, hypoalbuminemia ≤2.5 mg/dl, and edema, (2) age at disease onset less than 21 years. Patients with known monogenic NS or secondary NS due to identifiable underlying cause including infection, malignancy, medication, or other disease associated with NS such as systemic lupus erythematosus were excluded. Possible disease courses were classified according to the Kidney Disease: Improving Global Outcomes criteria and included SSNS (remission induced by standard corticosteroid therapy) and SRNS (defined as failure to enter remission after at least four to 6 weeks of standard corticosteroid therapy) (11). Patients for whom corticosteroid response category was unknown at time of data analysis (*n* = 125) were excluded from subcategory comparison analyses (SSNS vs. SRNS). The protocol Pro00014951 complies with the Declaration of Helsinki and the Declaration of Istanbul. All participants or guardians signed a written informed consent approved by Duke University's Institutional Review Board.

### Genotyping

Genotyping was performed for nine reported variants associated with childhood SSNS (6–10): *HLA-DQA1* [rs1129740], *BTNL2* [rs9348883], *HLA-DR/DQ* [rs4642516 & rs3134996], *Intergenic* (a locus between the genes *HLA-DQA1* and *HLA-DQB1*) [rs9273371], *CALHM6* [rs2637678], *NPHS1/KIRREL* [rs56117924], *TNFSF15* [rs6478109], and *TNFRSF11A* [rs34213471]. Variant details are provided in Supplementary Table S1. Information was gathered from dbSNP database and original reporting articles (6–10, 12). DNA samples were brought to a concentration of 50 ng/ul and genotyped using TaqMan or Direct Sanger Sequencing. TaqMan allele discrimination assay (Applied Biosystems, Foster City, CA) was carried out on an ViiA7 sequencer detector system according to the manufacturer's protocol. Quality control measures for genotyping included genotyping of duplicate samples as well as Centre d'Etude du Polymorphism Humain (CEPH) controls. To pass quality control, all duplicated samples had a match of 100%.

## Data analysis

### Allele frequency

We compared the risk allele frequencies (AF) between all patients with NS vs. controls and SRNS vs. controls from public databases (Genome Aggregation Database [gnomAD], Trans-Omics for Precision Medicine [TOPMed], and Allele Frequency Aggregator [ALFA]) (13–15). As the data for our cases have not been deposited in any public database, to the best of our knowledge, there is no overlap between cases and publicly available controls. Additionally, we compared AF between SSNS vs. SRNS. The groups were compared using odds ratios and chi square test of independence with Bonferroni correction for the number of tests in each set using R Statistical Software (16, 17).

### Variant burden analysis

**Figure 1:**
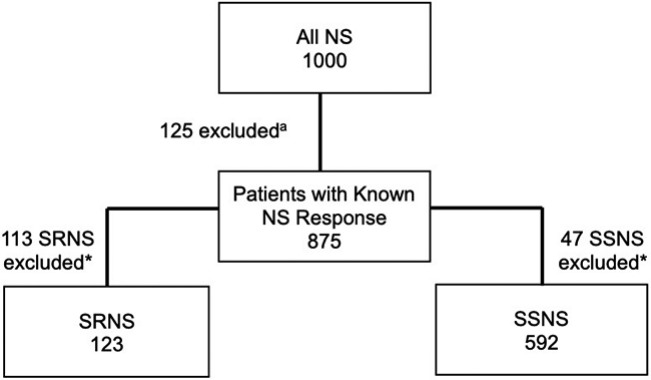
Flow diagram demonstrating exclusion of patients with missing phenotype^a^ and genotype* data for the known SSNS risk alleles (termed “missingness”, based on significantly different AF for each comparison group) for variant burden analysis. Number of patients excluded due to missingness: 113 patients with SRNS and 47 patients with SSNS.

To further characterize the role of these nine risk loci in pattern of therapy response, we investigated whether a higher burden of risk alleles was associated with increased odds of developing SSNS vs. SRNS. To determine the allele burden, the absolute number of risk alleles per patient was counted. Only variants with significant differences after Bonferroni correction in AF between therapy response groups were included in the variant burden analyses. For SSNS vs. SRNS, this included *HLA-DQA1* [rs1129740], *BTNL2* [rs9348883], *HLA-DR/DQ* [rs4642516 & rs3134996], *Intergenic* [rs9273371], and *CALHM6* [rs2637678]. Additionally, only individuals with no missing genetic data (“missingness”) across all variants used were included. Patients with missingness were excluded: 113 patients with SRNS, and 47 patients with SSNS (Figure 1). The number of risk alleles (variant burden) was tested for association with SSNS vs. SRNS using odds ratios and Fisher's exact test with Bonferroni correction for the number of tests in each set (17). We also compared the frequency of homozygous genotype for each risk variants between SSNS and SRNS groups.

## Results

### Patients

The total number of patients in our cohort was 1,000, which consisted of 639 patients with SSNS, 236 patients with SRNS, and 125 patients with unknown therapy response. Our cohort included self-reported race: Asian (38.4%), Black (28.8%), White (23.6%), Hispanic (6.0%), Mixed (2.0%), and Other (1.2%) (Table 1). The male-to-female ratio was 2:1 (62.7% vs. 36.7%). Median age at disease onset was 4.0 years (IQR 4.9). Almost half of the patients with SRNS were Black (45.3%) and SRNS patients were older at diagnosis (median age 8.0 years, IQR 9.8) (Table 1).

**Table 1:** Demographic characteristics of the study population.

	SSNS (*n* = 639)	SRNS (*n* = 236)	Unknown^b^ (*n* = 125)	Overall (*n* = 1,000)
Age^a^ (years)				
Mean (SD)	4.6 (3.2)	8.3 (5.4)	5.4 (4)	5.5 (4.2)
Median (IQR)	3.3 (3.8)	8.0 (9.8)	4.0 (4.8)	4.0 (4.9)
Missing *n*, (%)	6 (1)	22 (9.3)	33 (26.4)	61 (6.1)
Sex (%)				
Female	223 (34.9)	99 (42.0)	45 (36.0)	367 (36.7)
Male	414 (64.8)	135 (57.2)	78 (62.4)	627 (62.7)
Missing	2 (0.3)	2 (0.8)	2 (1.6)	6 (0.6)
Race (%)				
White	124 (19.4)	78 (33.1)	34 (27.2)	236 (23.6)
Black	134 (21.0)	107 (45.3)	47 (37.6)	288 (28.8)
Asian	344 (53.8)	20 (8.5)	20 (16.0)	384 (38.4)
Hispanic	23 (3.6)	25 (10.6)	12 (9.6)	60 (6.0)
Mixed	12 (1.9)	5 (2.1)	3 (2.4)	20 (2.0)
Other	2 (0.3)	1 (0.4)	9 (7.2)	12 (1.2)

SSNS, steroid sensitive nephrotic syndrome; SRNS, steroid resistant nephrotic syndrome.

^a^ Age refers to age at diagnosis.

^b^ Unknown refers to unknown pattern of response to standard corticosteroid therapy at time of data analysis.

### Frequency of NS variants

Risk AF was compared for each risk loci between NS vs. controls (Table 2) and SSNS vs. SRNS (Table 3).

**Table 2:** Allele frequency for risk variants in nephrotic syndrome (NS) vs. controls.

SNP	Risk allele	Gene	MAF published controls^b^	Risk AF: controls risk/total (frequency)	Risk AF: NS cases risk/total (frequency)	OR (95% CI)	*p*-value^a^
rs1129740	A	*HLA-DQA1*	*A* = 0.49 (6)	94,628/191,328 (0.50)	1,251/1,784 (0.70)	2.4 (2.2–2.7)	<2.2 × 10^−16^*
rs9348883	A	*BTNL2*	*T* = 0.94 (7)	15,739/264,690 (0.06)	330/1,756 (0.19)	3.7 (3.2–4.1)	<2.2 × 10^−16^*
rs4642516	T	*HLA-DR/DQ*	*G* = 0.27 (9)	138,950/264,690 (0.52)	1,211/1,752 (0.69)	2.0 (1.8–2.2)	<2.2 × 10^−16^*
rs3134996	A	*HLA-DR/DQ*	*A* = 0.28 (9)	94,736/264,690 (0.36)	1,494/1,762 (0.85)	10.0 (8.8–11.4)	<2.2 × 10^−16^*
rs9273371	T	*Intergenic*	NP (8)	67,317/264,690 (0.25)	736/1,764 (0.42)	2.1 (1.9–2.3)	<2.2 × 10^−16^*
rs2637678	T	*CALHM6*	*C* = 0.40 (8)	167,819/264,690 (0.63)	1,329/1,766 (0.75)	1.8 (1.6–2.0)	<2.2 × 10^−16^*
rs56117924	A	*NPHS1/KIRREL*	*A* = 0.17 (10)	14,543/264,690 (0.06)	125/1,764 (0.07)	1.3 (1.1–1.6)	3.5 × 10^−3^*
rs6478109	G	*TNFSF15*	*A* = 0.39 (10)	224,655/328,718 (0.68)	1,393/1,762 (0.79)	1.7 (1.6–2.0)	<2.2 × 10^−16^*
rs34213471	A	*TNFRSF11A*	*A* = 0.31 (10)	14,311/264,690 (0.05)	207/1,766 (0.12)	2.3 (2.0–2.7)	<2.2 × 10^−16^*

SNP, single nucleotide polymorphism; NP, not published.

* Significance after Bonferroni correction with *α* = 0.05.

^a^ *P*-values from chi square test of independence.

^b^ References for MAF published data controls are next to each AF.

**Table 3:** Allele frequency for risk variants SSNS vs. SRNS.

SNP	Risk allele	Gene	Risk AF: SSNS risk/total (frequency)	Risk AF: SRNS risk/total (frequency)	Odds ratio (95% CI)	*p*-value^a^
rs1129740	G	*HLA-DQA1*	333/1,228 (0.27)	150/326 (0.46)	2.3 (1.8–2.9)	5.7 × 10^−11^*
rs9348883	A	*BTNL2*	125/1,198 (0.10)	184/342 (0.54)	10.0 (7.5–13.3)	<2.2 × 10^−16^*
rs4642516	G	*HLA-DR/DQ*	339/1,194 (0.28)	145/346 (0.42)	1.8 (1.4–2.3)	1.9 × 10^−6^*
rs3134996	T	*HLA-DR/DQ*	147/1,196 (0.12)	96/352 (0.27)	2.7 (2.0–3.5)	1.1 × 10^−11^*
rs9273371	C	*Intergenic*	685/1,198 (0.57)	231/350 (0.66)	1.5 (1.1–1.9)	3.1 × 10^−3^*
rs2637678	C	*CALHM6*	266/1,198 (0.22)	124/352 (0.35)	1.9 (1.5–2.5)	8.7 × 10^−7^*
rs56117924	A	*NPHS1/KIRREL*	80/1,198 (0.07)	24/352 (0.07)	1.0 (0.6–1.6)	0.9
rs6478109	A	*TNFSF15*	244/1,198 (0.20)	82/350 (0.23)	1.2 (0.9–1.6)	0.2
rs34213471	C	*TNFRSF11A*	1,034/1,198 (0.80)	322/352 (0.91)	1.7 (1.1–2.6)	0.01

SNP, single nucleotide polymorphism.

^a^ *P*-values from chi square test of independence.

* Significance after Bonferroni correction with *α* = 0.05.

### AF comparison for NS vs. controls

To validate the association between the known SSNS risk loci and patients with childhood-onset NS, we compared AF between all patients in our cohort with NS to AF found in public databases as mentioned previously (Table 2). AF of all risk loci were significantly different between all patients with NS compared with controls with *p* < 3.5 × 10^−3^. When we specifically compared SRNS vs. controls, four risk alleles (*BTNL2* [rs9348883]*, HLA-DR/DQ* [rs3134996], intergenic [rs9273371], *and TNFSF15* [rs6478109]) remained significantly different (Supplementary Table S2).

### AF comparison for SSNS vs. SRNS

To evaluate our hypothesis that known NS risk loci are associated with corticosteroid therapy response in patients with childhood-onset NS, we compared the AF between SSNS vs. SRNS (Table 3). Six of the reported risk alleles had significantly different AF when comparing SSNS vs. SRNS: *HLA-DQA1* [rs1129740], *BTNL2* [rs9348883], *HLA-DR/DQ* [rs4642516 & rs3134996], *Intergenic* [rs9273371], *CALHM6* [rs2637678] (*p* < 3.1 × 10^−3^). Three risk alleles (*NPHS1/KIRREL* [rs56117924], *TNFSF15* [rs6478109], and *TNFRSF11A* [rs34213471]) did not have significantly different AF between SSNS and SRNS after Bonferroni correction, indicating that *NPHS1/KIRREL,TNFSF15 and TNFRSF11A* are not associated with therapy response when comparing SSNS vs. SRNS.

### Risk variant burden analysis

**Figure 2:**
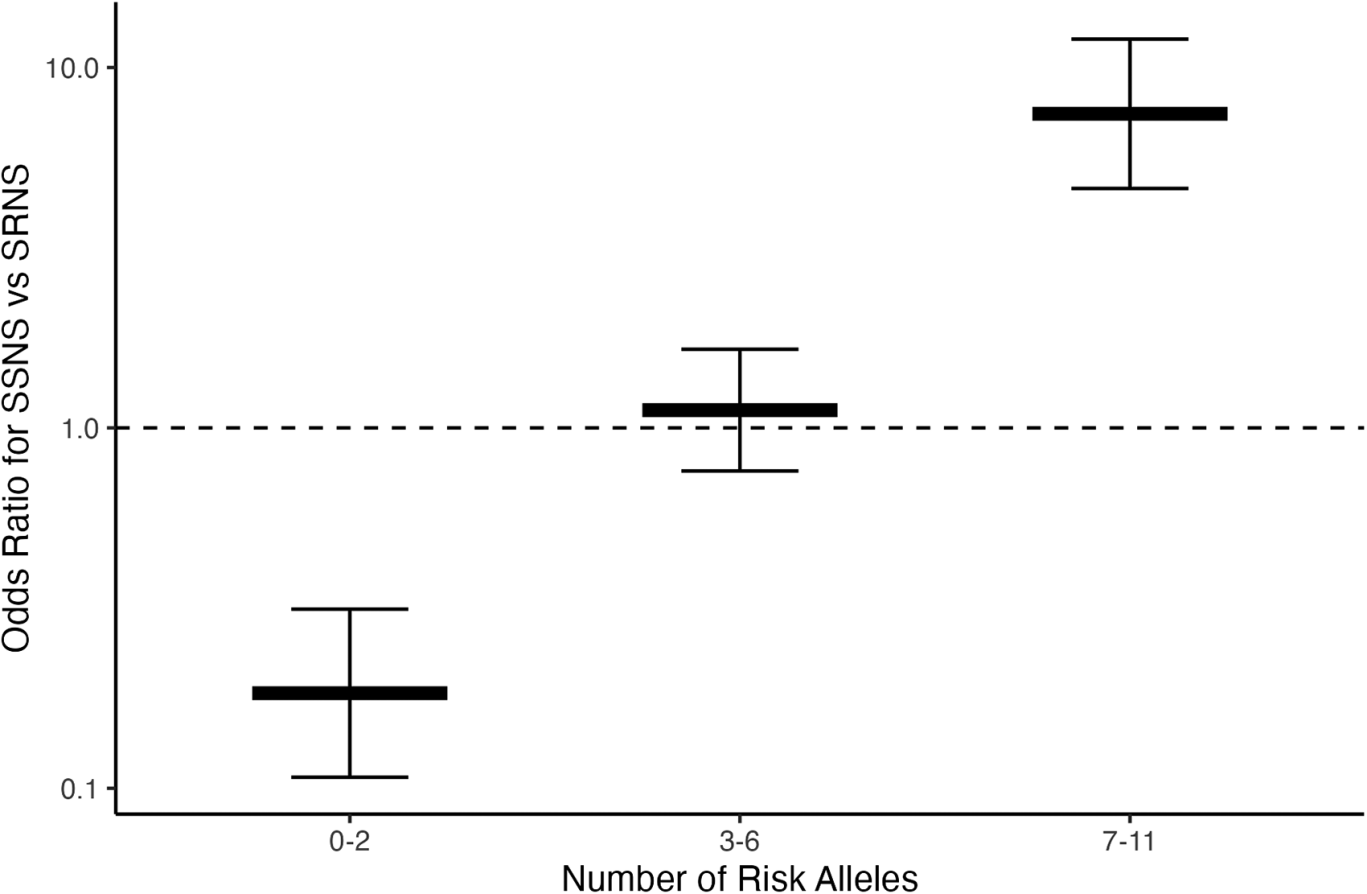
Risk loci burden for patients with SSNS vs. SRNS. NS patients with ≥7 risk alleles have significantly increased odds of developing SRNS (OR 7.4, 95% CI 4.6–12.0; *p* = 8.2 × 10^−16^). Note, there were no patients with 12 alleles.

To assess whether risk variant burden is associated with pattern of corticosteroid therapy response in childhood NS, we included only those risk alleles found to have a significantly different AF after Bonferroni correction between SSNS vs. SRNS (Table 3). Additionally, we only included individuals with no missingness, thus, 47 and 113 patients were excluded from SSNS and SRNS group respectively (Figure 1). Patients with risk allele burden ≥7 had significantly greater odds of developing SRNS (OR 7.4, 95% CI 4.6–12.0; *p* = 8.2 × 10^−16^, Figure 2 and Supplementary Table S3). Allele burden was grouped based on patterns determined from ungrouped counts as shown in Supplementary Table S4 and Figure S1 for SSNS vs. SRNS. As expected, when comparing the frequency of homozygous variants between SSNS and SRNS group, we found that SRNS patients were enriched for homozygous variants (Supplementary Table S5).

## Discussion

There are currently no robust clinical, laboratory, or genetic predictors of pattern of therapy response in patients with NS. Factors such as age at presentation, serum albumin, hematuria, and total duration of corticosteroid therapy have been studied, but the results are often inconsistent and not reproducible (18–20). Therefore, in this study, we investigated the relationship between known SSNS risk alleles from unbiased GWAS studies and therapy response. Our study confirmed that the known SSNS risk loci are indeed associated with all types of NS compared with historical controls. Importantly, we are the first to demonstrate that the variant burden of six of these nine risk alleles is associated with increased odds of having SRNS vs. SSNS, the most severe form of NS. Thus, these six risk loci may be helpful tools to add to the clinical armamentarium for predicting pattern of response.

Herein, we validated nine previously identified genetic risk variants for the development of childhood-onset NS (6–10).These risk variants are in genes associated with adaptive immunity (*HLA-DQA1, BTNL2*, *HLA-DR/DQ*, *CALHM6*, *TNFSF15,* and *TNFRSF11A)* and maintenance of podocyte structure and function (*NPHS1/KIRREL).* While these variants have been associated with childhood SSNS and were individually identified through GWAS studies, they have not yet been studied as a group of risk factors in patients with all types of childhood NS (SSNS and SRNS). Importantly, we showed that a subgroup of variants in *BTNL2, HLA-DR/DQ, Intergenic, and TNFSF15* were also associated with SRNS compared to controls and may be able to differentiate SSNS from SRNS. Prospective studies, however, are required prior to their widespread use in clinical practice.

In our study, the *HLA-DQA1* [rs1129740], *HLA-DR/DQ* [rs3134996], and an Intergenic locus between *HLA-DQA1* and *HLA-DQB1* [rs9273371] had significantly different AF in NS vs. controls, confirming that these alleles are associated with childhood NS. It has been proposed that variants in the *HLA* genes lead to inappropriate antigen stimulation and results in abnormal T-lymphocyte function, possibly leading to podocyte injury (6).

Additionally, we studied *BTLN2* risk locus in our multi-race cohort and found the AF was significantly different in patients with NS vs. controls as well as when comparing SSNS vs. SRNS patients, suggesting it is associated with disease and therapy response. The BTNL2 protein is a type-1 membrane protein which provides co-inhibitory signals to T-lymphocytes (21). *BTNL2* also induces FoxP3 expression and development of regulatory T-cells (22).

Furthermore, the *CALHM6* risk locus [rs2637678] had a significantly different AF in NS vs. controls and SSNS vs. SRNS, suggesting that this allele is associated with both disease and therapy response. *CALHM6* is typically expressed in immune cells, including B- and T-lymphocytes, NK cells, and macrophages and encodes for a cation channel important in the transport of various molecules between cells, signifying that CALHM6 proteins are important for immune cell interactions (23). It is speculated that some members of the CALHM6 family regulate apoptosis of immune cells, and dysfunction of these proteins leads to the impaired immune regulation through multiple pathways leading to development of NS (8).

We also demonstrated that the TNF gene family yet again played an important role in NS. Jia et al. previously identified a risk variant in the *TNFSF15* gene [rs6478109] in their Japanese cohort which was replicated in Korean, South Asian, and African populations (10). The *TNFSF15* (Tumor Necrosis Factor Super-Family Member 15) gene encodes for the ligand TL1A (Tumor Necrosis Factor-Like Cytokine 1A), which is expressed on antigen presenting cells and a variety of lymphocyte subtypes (24). The activation of TL1A plays important roles in chronic immune-mediated diseases and has been demonstrated in kidney-specific disease such as ANCA-associated renal vasculitis (24, 25). Interestingly, the risk allele at the *TNFSF15* locus [rs6478109] had significantly different AF in patients with NS vs. cases in our cohort but did not have a significantly different AF when comparing corticosteroid therapy response (SSNS vs. SRNS), suggesting this allele is associated with NS but not a predictor of therapy response.

A second variant was identified by Jia et al. in the Tumor Necrosis Factor Receptor Super-Family Member 11A (*TNFRSF11A*) region (10). T-lymphocytes are known to express RANKL (Receptor Activator of NF- κB Ligand) and to act on dendritic cells which are a major antigen presenting cell (26). In addition, the Receptor Activator of NF- κB (RANK) + RANKL signaling is important in the negative selection during T-lymphocyte development (27). In the present study, the risk allele for this locus was found to have a significantly different AF in NS cases vs. controls, indicating association with disease.

Finally, highly penetrant pathogenic variants in *NPHS1* are known causes of monogenic nephrotic syndrome. This is interesting, as previously reported and in the present study, common variants in this same gene are risk factors for NS. More recently, autoantibodies to nephrin have been reported to be associated with minimal change disease (MCD), the clinicopathologic correlate of SSNS. It is unclear if the variants in *NPHS1* in the presence of permissive *HLA* variants are the drivers of autoantibody production against nephrin (28). Further studies to examine the relationship between *NPHS1* and *HLA* variants with nephrin autoantibodies are warranted, in light of our findings.

Beside validating these nine genetic risk variants, we more importantly are the first to demonstrate the variant burden for corticosteroid therapy response in a large, multi-race cohort. As the variant burden increased, the severity of NS potentially increased. Our findings validate and expand on the work of Debiec and colleagues who found that having a higher variant burden, specifically having three or four of *HLA-D* risk alleles (rs1063348 and rs28366266) was associated with disease course in patients with NS. Variant burden has been studied in several other immune-mediated diseases in both additive and non-additive manners and have been found to have similar results with variant burden explaining phenotypic variance (29, 30).

Beyond predicting disease development, identifying ways to predict response to traditional therapies is important. The use of immunomodulatory therapy should not be taken lightly as these medications have potentially serious side effects. For NS in particular, corticosteroid therapy is the treatment of choice for the initial presentation (31). Therefore, children are begun on this therapy and response is monitored (namely, resolution of proteinuria). Most patients respond well to corticosteroids (32). However, a subset of patients will not respond to corticosteroid therapy (i.e., will not experience remission of proteinuria, termed SRNS), therefore identification of predictors of therapy response will avoid unnecessary exposure of this subgroup to major side effects of corticosteroid.

While clinical factors have not been shown to definitively predict disease course or development, risk prediction scores similar to polygenic risk scores for cardiovascular disease have been proposed for childhood SSNS (33–35). Ideally, combining clinical factors at diagnosis with genetic testing for both risk variants burden and monogenic causes of NS could be developed into a similar risk score to predict corticosteroid therapy response in patients with childhood-onset NS in order to mitigate harmful side effects of prolonged corticosteroid exposure and streamline to more targeted B and T cell therapies.

Although our study demonstrated promising findings, limitations of this study exist. One such limitation included a lack of racially matched controls. We attempted to mitigate this effect by choosing control data from public databases containing genetic information from a multitude of studies conducted worldwide (13–15). Additionally, we relied on self-reported race and our sample size was suboptimal for risk stratification by race. Moreover, as this was a retrospective analysis, we did not have intermediate outcomes such as frequent relapsing, steroid dependent, and late steroid responsiveness. Finally, genotyping for *APOL1* CKD risk genotype was not available for our cohort. However, previous studies have shown that the *APOL1* high-risk genotype is strongly associated with SRNS but not SSNS in individuals of African ancestry (36).

In conclusion, our study confirmed that certain genetic risk variants are associated with NS as well as pattern of corticosteroid therapy response in patients with childhood-onset NS. We determined that variant burden of these risk loci is associated with pattern of therapy response. This data suggests that this six variant panel may be a promising tool to determine corticosteroid response and guide NS management. Future prospective studies are needed to confirm these observations and investigate the mechanisms by which each risk loci contributes to disease development and therapy response.

## Data Availability

The datasets generated and/or analyzed during the current study are not publicly available due to the risk of patient identification through genotype level data. However, data are available from the corresponding author upon reasonable request.
