# Supplementary material for: Genetic risk variants for childhood nephrotic syndrome and corticosteroid response
Source: Front Pediatr. 2023 Oct 6;11:1248733. doi: 10.3389/fped.2023.1248733 (PMC10588181; doi:10.3389/fped.2023.1248733)
Supplement: Supplementary file 1 [file Datasheet1.pdf]

Supplementary Material

Genetic variants for childhood nephrotic syndrome and corticosteroid therapy response

Rachel K. Cason, Eileen Chambers, Tiffany Tu, Megan Chryst-Stangl, Kinsie Huggins, Brandon M. Lane, Alejandro Ochoa, Annette Jackson, Rasheed A. Gbadegesin*

*** Correspondence:** Rasheed Gbadegesin: rasheed.gbadegesin@duke.edu

1 Supplementary Figures and Tables

Supplementary Table 1 NS Allele Descriptions

SNP	Gene	Chromosome	NS Risk Allele	Position	Type
rs1129740	HLA-DQA1	6	A	32609103	Missense C34Y
rs9348883	BTNL2	6	A	32358549	Intronic variant
rs4642516	HLA-DR/DQ	6	T	32657543	Intergenic
rs3134996	HLA-DR/DQ	6	A	32636866	Intergenic
rs9273371	Intergenic	6	T	32626565	Intergenic
rs2637678	CALHM6	6	T	116787378	Intergenic
rs56117924	NPHS1/KIRREL	19	A	36334182	Intronic variant
rs6478109	TFNSF15	9	G	117568766	Upstream transcript variant
rs34213471	TNFRSF11A	18	A	60008436	Intronic variant

Supplementary Table 2 Risk allele frequency for SRNS vs. Controls: [a] P-values from chi square test of independence. *Significance after Bonferroni correction with $\alpha = 0.05$. SNP, single nucleotide polymorphism.

SNP	Risk Allele	Gene	Risk AF: Controls Risk/Total (frequency)	Risk AF: SRNS Risk/Total (frequency)	Odds Ratio (95% CI)	P-value[a]
rs1129740	A	*HLA-DQA1*	94628/191328 (0.50)	176/326 (0.54)	1.2 (1.0-1.5)	0.1
rs9348883	A	*BTNL2*	15739/264690 (0.06)	184/342 (0.54)	18.3 (14.9-22.8)	$<2.2 \times 10^{-16}$*
rs4642516	T	*HLA-DR/DQ*	138950/264690 (0.52)	201/346 (0.58)	1.3 (1.0-1.6)	0.04
rs3134996	A	*HLA-DR/DQ*	94736/264690 (0.36)	256/352 (0.73)	4.8 (3.8-6.0)	$<2.2 \times 10^{-16}$*
rs9273371	T	*Intergenic*	67317/264690 (0.25)	119/350 (0.34)	1.5 (1.2-1.9)	2.4×10^{-4}*
rs2637678	T	*CALHM6*	167819/264690 (0.63)	229/352 (0.65)	1.1 (0.9-1.3)	0.5
rs56117924	A	*NPHS1/KIRREL*	14543/264690 (0.06)	24/352 (0.07)	1.3 (0.8-1.9)	0.3
rs6478109	G	*TNFSF15*	224655/328718 (0.68)	268/350 (0.77)	1.5 (1.2-1.9)	9.4×10^{-4}*
rs34213471	A	*TNFRSF11A*	14311/264690 (0.05)	30/352 (0.09)	1.6 (1.1 to 2.4)	9.8×10^{-3}

Supplementary Table 3 Odds ratio risk analysis for SSNS vs. SRNS: [a] P-values from Fisher exact test; *significance after Bonferroni correction with α= 0.05.

# Risk Alleles	# SRNS	# SSNS	Total SRNS	Total SSNS	OR	Lower CI	Upper CI	P-value[a]
0-2	17	276	123	592	0.2	0.1	0.3	2.1×10^{-12}*
3-6	60	272	123	592	1.1	0.8	1.7	0.6
7-11	46	44	123	592	7.4	4.6	12.0	8.2×10^{-16}*

Supplementary Table 4 Odds ratio risk analysis for SSNS vs. SRNS with ungrouped counts: [a] P-values from Fisher exact test; *significance after Bonferroni correction with α= 0.05

# Risk Alleles	# SRNS	# SSNS	Total SRNS	Total SSNS	OR	Lower CI	Upper CI	P-value[a]
0	1	17	123	592	0.3	0.04	2.1	0.3
1	6	126	123	592	0.2	0.1	0.4	$3.0x10^{-6}$*
2	10	133	123	592	0.3	0.2	0.6	$1.7x10^{-4}$*
3	24	106	123	592	1.1	0.7	1.8	0.7
4	18	66	123	592	1.4	0.8	2.4	0.3
5	10	64	123	592	0.7	0.4	1.5	0.4
6	8	36	123	592	1.1	0.5	2.4	0.8
7	18	17	123	592	5.8	2.9	11.6	$1.9x10^{-6}$*
8	11	16	123	592	3.5	1.6	7.8	$2.8x10^{-3}$*
9	7	9	123	592	3.9	1.4	10.7	$1.1x10^{-2}$
10	8	2	123	592	20.5	4.3	97.9	$2.1x10^{-5}$*
11	2	0	123	592	Inf	NA	Inf	$3.0 x 10^{-2}$
12	0	0	123	592	NA	NA	NA	1.0

Supplementary Table 5 Frequency of homozygous patients for SSNS vs. SRNS risk alleles: [a]P-value calculated using Fisher exact test. *Significance after Bonferroni correction with α= 0.05. SNP, single nucleotide polymorphism.

**SNP (gene)**	**Risk Allele**	**#homozygous SSNS /total SSNS patients (frequency)**	**#homozygous SRNS /total SRNS patients (frequency)**	**Odds Ratio (95% CI)**	**P-value[a]**
rs1129740 (*HLA-DQA1)*	G	71/614 (0.12)	68/163 (0.42)	5.46 (3.6-8.3)	<2.2x10^{-16}*
rs9348883 *(BTNL2)*	A	7/599 (0.01)	18/171 (0.11)	9.9 (3.9-28.6)	8.9x10^{-8}*
rs4642516 *(HLA-DR/DQ)*	G	51/597 (0.09)	35/173 (0.20)	2.4 (1.4-3.8)	4.7x10^{-4}*
rs3134996 *(HLA-DR/DQ)*	T	16/598 (0.03)	15/176 (0.09)	3.4 (1.5-7.5)	1.5x10^{-3}*
rs9273371 *(intergenic)*	C	206/599 (0.34)	78/175 (0.45)	1.5 (1.1-2.2)	0.02
rs2637678 *(CALHM6)*	C	25/299 (0.04)	22/176 (0.125)	3.3 (1.7-6.2)	2.0x10^{-4}*
rs56117924 *(NPHS1/KIRREL)*	A	3/599 (0.005)	0/176 (0)	0 (0-8.25)	1
rs6478109 *(TNFSF15)*	A	27/599 (0.05)	11/175 (0.06)	1.4 (0.6-3.0)	0.3
rs34213471 *(TNFRSF11A)*	C	450/599 (0.75)	147/176 (0.84)	1.7 (1.1-2.7)	0.02

1.1 Supplementary Figures

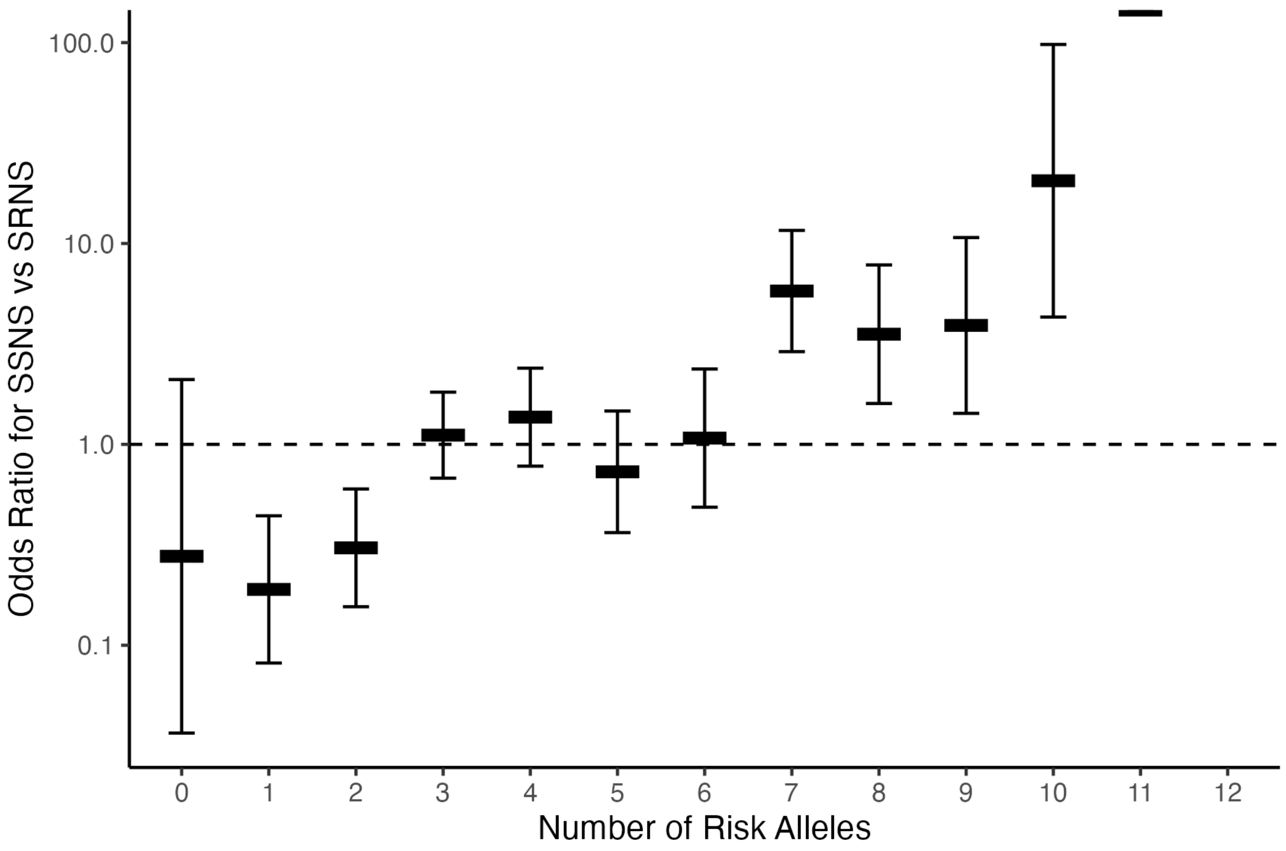

Supplementary Figure 1 Risk loci burden for patients with SSNS vs. SRNS. Odds ratio for allele burden between SSNS vs. SRNS with ungrouped counts
